# Gastric bypass alters diurnal feeding behavior and reprograms the hepatic clock to regulate endogenous glucose flux

**DOI:** 10.1172/jci.insight.166618

**Published:** 2023-03-22

**Authors:** Yuanchao Ye, Marwa Abu El Haija, Reine Obeid, Hussein Herz, Liping Tian, Benjamin Linden, Yi Chu, Deng Fu Guo, Daniel C. Levine, Jonathan Cedernaes, Kamal Rahmouni, Joseph Bass, Mohamad Mokadem

**Affiliations:** 1 Department of Internal Medicine and; 2 Stead Family Department of Pediatrics, University of Iowa Carver College of Medicine, Iowa City, Iowa, USA.; 3 Department of Pediatrics, Division of Gastroenterology, Hepatology & Nutrition, Stanford University School of Medicine, Palo Alto, California, USA.; 4 Department of Biology, American University of Beirut, Beirut, Lebanon.; 5 Department of Clinical Pharmacy, School of Basic Medical Sciences and Clinical Pharmacy, China Pharmaceutical University, Nanjing, China.; 6 Department of Neuroscience and Pharmacology, University of Iowa Carver College of Medicine, Iowa City, Iowa, USA.; 7 VA Iowa City Healthcare System, Iowa City, Iowa, USA.; 8 Department of Medicine, Feinberg School of Medicine, Northwestern University, Chicago, Illinois, USA.; 9 Fraternal Order of Eagles Diabetes Research Center and; 10 Obesity Research & Education Initiative, University of Iowa, Iowa City, Iowa, USA.

**Keywords:** Endocrinology, Metabolism, Gluconeogenesis, Glucose metabolism

## Abstract

The molecular clock machinery regulates several homeostatic rhythms, including glucose metabolism. We previously demonstrated that Roux-en-Y gastric bypass (RYGB) has a weight-independent effect on glucose homeostasis and transiently reduces food intake. In this study we investigate the effects of RYGB on diurnal eating behavior as well as on the molecular clock and this clock’s requirement for the metabolic effects of this bariatric procedure in obese mice. We find that RYGB reversed the high-fat diet–induced disruption in diurnal eating pattern during the early postsurgery phase of food reduction. Dark-cycle pair-feeding experiments improved glucose tolerance to the level of bypass-operated animals during the physiologic fasting phase (Zeitgeber time 2, ZT2) but not the feeding phase (ZT14). Using a clock gene reporter mouse model (*mPer2^Luc^*), we reveal that RYGB induced a liver-specific phase shift in peripheral clock oscillation with no changes to the central clock activity within the suprachiasmatic nucleus. In addition, we show that weight loss effects were attenuated in obese *Clock*^Δ19^ mutant mice after RYGB that also failed to improve glucose metabolism after surgery, specifically hepatic glucose production. We conclude that RYGB reprograms the peripheral clock within the liver early after surgery to alter diurnal eating behavior and regulate hepatic glucose flux.

## Graphical abstract

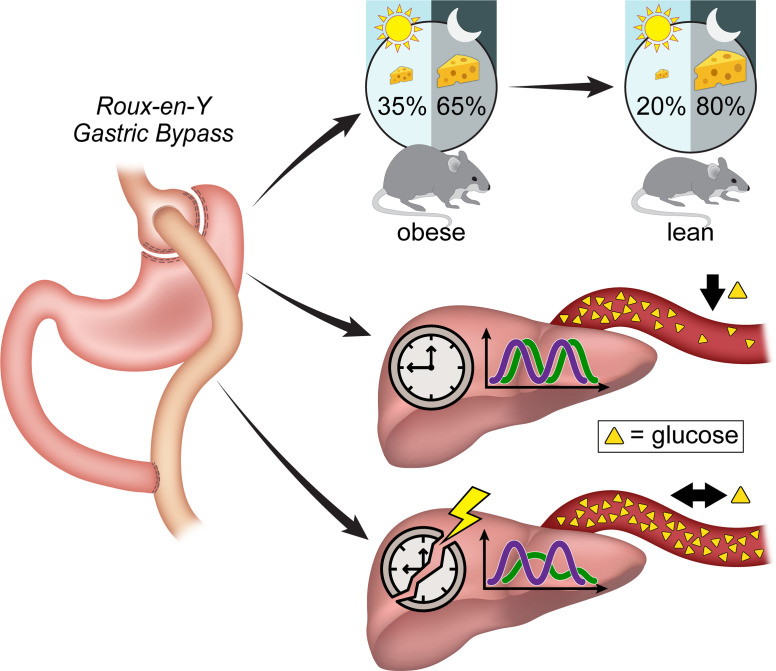

## Introduction

Rhythmic body functions are orchestrated by the molecular clock machinery, which consists of a transcriptional-translational feedback loop of transcription factors (Clock: basic helix-loop-helix ARNT like 1 [Bmal1], Period 1–3, Cryptochrome 1–2) that regulate their own rhythmicity as well as that of other downstream genes that regulate metabolism (1, 2). The central clock, which is present within the suprachiasmatic nucleus (SCN), is mainly entrained by light and synchronizes physiology and behavior to the environmental light/dark cycle. Outside the SCN, the peripheral clock is expressed in every mammalian cell, including the liver, where it is mainly entrained by food or energy availability, enabling synchronization of energy metabolism to fast–sleep/feed–wake cycles (3–5). Moreover, it was shown that the liver clock is necessary and sufficient to synchronize the rhythmicity of other peripheral clock tissues to feeding-related signals (6). Environmental or genetic disruption in circadian rhythmicity promotes insulin resistance, weight gain, and type 2 diabetes (7–11).

Bariatric surgery is now recognized to have an important role in management of morbid obesity and uncontrolled type 2 diabetes, as it confers long-term protection from cardiovascular complications, compared with intensive medical therapy (12, 13). In obese animal models, it was previously demonstrated that Roux-en-Y gastric bypass (RYGB) exerts a weight-independent effect on glucose homeostasis (14, 15). We, and others, have consistently found that RYGB induces an increase in energy expenditure and a transient reduction in 24-hour food intake. However, no comprehensive investigation has been undertaken to examine circadian feeding behavior after bypass surgery (14–19). Interestingly, vertical sleeve gastrectomy (VSG), another common bariatric procedure, was shown to exert the same weight loss, food reduction, and glucose regulatory effects in *Clock*^Δ19^ mutant mice (homozygous deficiency in functional *Clock* gene expression controlling core clock machinery) as wild-type (WT) littermates (20). In addition, VSG does not seem to significantly alter either circadian eating behavior or clock expression, within the SCN or any of the peripheral tissues, except for the subcutaneous white adipose tissue (WAT) (20, 21).

Despite the initial similarities in weight loss response, gastric bypass has significant anatomical modifications that distinguish it from VSG. We recently described key differences between RYGB and VSG regarding their underlying molecular mechanisms for energy regulation (19), where RYGB, but not VSG, engages the peripheral cannabinoid system to increase splanchnic nerve activity and induce thermogenesis in visceral mesenteric fat. Here, we aimed to examine the effects of RYGB on circadian feeding behavior, as well as clock gene rhythms in multiple metabolic tissues. Furthermore, we performed RYGB in *Clock*^Δ19^ mutant mice to explore the involvement of the molecular clock machinery in the metabolic effects of RYGB.

## Results

### RYGB transiently reverses the high-fat diet–induced alteration in the diurnal feeding pattern.

**Figure 1:**
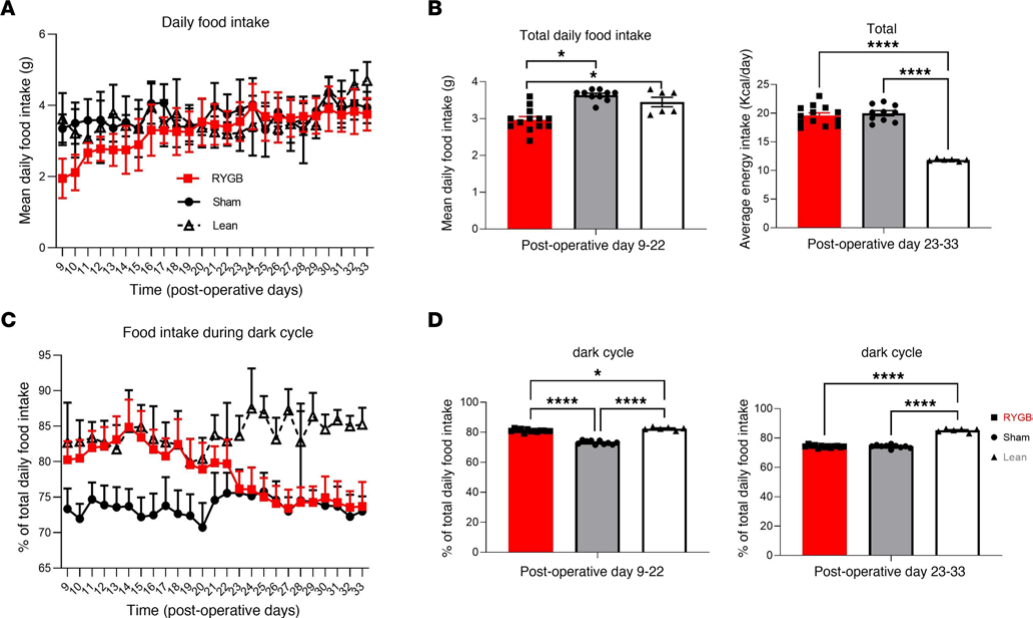
RYGB transiently reverses the HFD-induced alteration in the diurnal feeding pattern. (**A**) Average daily food intake (g) in RYGB and Sham-operated C57BL/6J mice fed 60% HFD and age-matched lean counterparts fed regular chow over several weeks postsurgery. (**B**) Average daily energy intake (kcal/g) among all 3 intervention groups during the first 2 weeks postsurgery compared with the second 2 weeks postsurgery. (**C**) Dark-cycle food intake expressed as percentage of total daily intake in RYGB and Sham-operated C57BL/6J mice fed 60% HFD and age-matched lean counterparts fed regular chow over several weeks (shown as days) postsurgery. (**D**) Average dark-cycle food intake (expressed as % of total intake) among all 3 intervention groups, during the first 2 weeks postsurgery, compared with the second 2 weeks postsurgery. Mean ± SEM. *N*, RYGB = 13, Sham = 10, Lean = 6. One-way ANOVA followed by Tukey’s test. *P < 0.05, ****P < 0.001.

Diet-induced obesity (DIO) mice were placed on their preoperative high-fat diet (HFD) on day 8 after recovery from surgery. RYGB-operated mice showed a decrease in their daily (24-hour) food intake for approximately 2 weeks (days 9–22) compared with their sham-operated counterparts (Figure 1, A and B), after which the average daily and cumulative food intake was similar between the 2 groups. Percentage of food intake during the dark cycle was increased in the RYGB-operated mice to the level of age-matched lean mice that were fed regular chow diet during this transient period of decrease in energy consumption (Figure 1, C and D).

### Pair-feeding to RYGB mice improves glucose tolerance during the fasting cycle.

**Figure 2:**
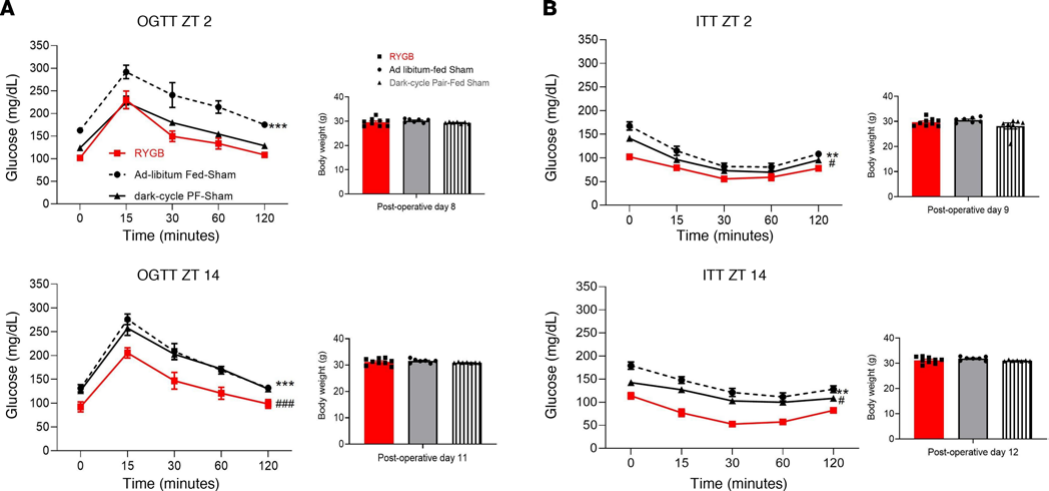
Pair-feeding to RYGB counterparts improves glucose tolerance during the light/fasting cycle. (**A**) Oral glucose tolerance test and (**B**) insulin tolerance test performed during postoperative week 2 (day 8–12) at Zeitgeber time 2 (ZT2) and ZT14 for RYGB, ad libitum–fed Sham and dark-cycle pair-fed (PF) Sham. Average body weight (g) before each experiment is listed below its corresponding graph. RYGB, *n* = 9, ad libitum–fed Sham, *n* = 7, dark-cycle PF Sham, *n* = 7. Mean ± SEM. Two-way ANOVA followed by Tukey’s test. **P < 0.01, ***P < 0.001 for ad libitum–fed Sham versus RYGB; ^#^*P* < 0.05, ^###^*P* < 0.001 for dark-cycle PF Sham versus RYGB.

When pair-fed (PF) during the physiologic eating diurnal cycle, these dark-cycle PF sham mice gained less weight than their ad libitum–fed counterparts in a 4-week period yet significantly more than RYGB mice (Supplemental Figure 1, A and B; supplemental material available online with this article; https://doi.org/10.1172/jci.insight.166618DS1). Oral glucose tolerance tests and insulin tolerance tests (OGTTs and ITTs) were performed during week 2 (when body weights were similar) in dark-cycle PF sham and RYGB mice at 2 time points: Zeitgeber time 2 (ZT2; fasting phase) and ZT14 (feeding phase). Interestingly, dark-cycle PF sham mice exhibited similar glucose tolerance to RYGB mice during the fasting phase as shown by the OGTT curves of PF shams looking like the RYGB mice during ZT2 (fasting phase) but looking like the ad libitum–fed shams during ZT14 (feeding phase) (Figure 2A). On the other hand, dark-cycle PF sham mice showed an intermediate insulin sensitivity phenotype relative to RYGB and ad libitum–fed sham mice during ZT2 and ZT14 (Figure 2B).

### RYGB induces a phase shift in hepatic clock gene oscillation but not in the SCN.

**Figure 3:**
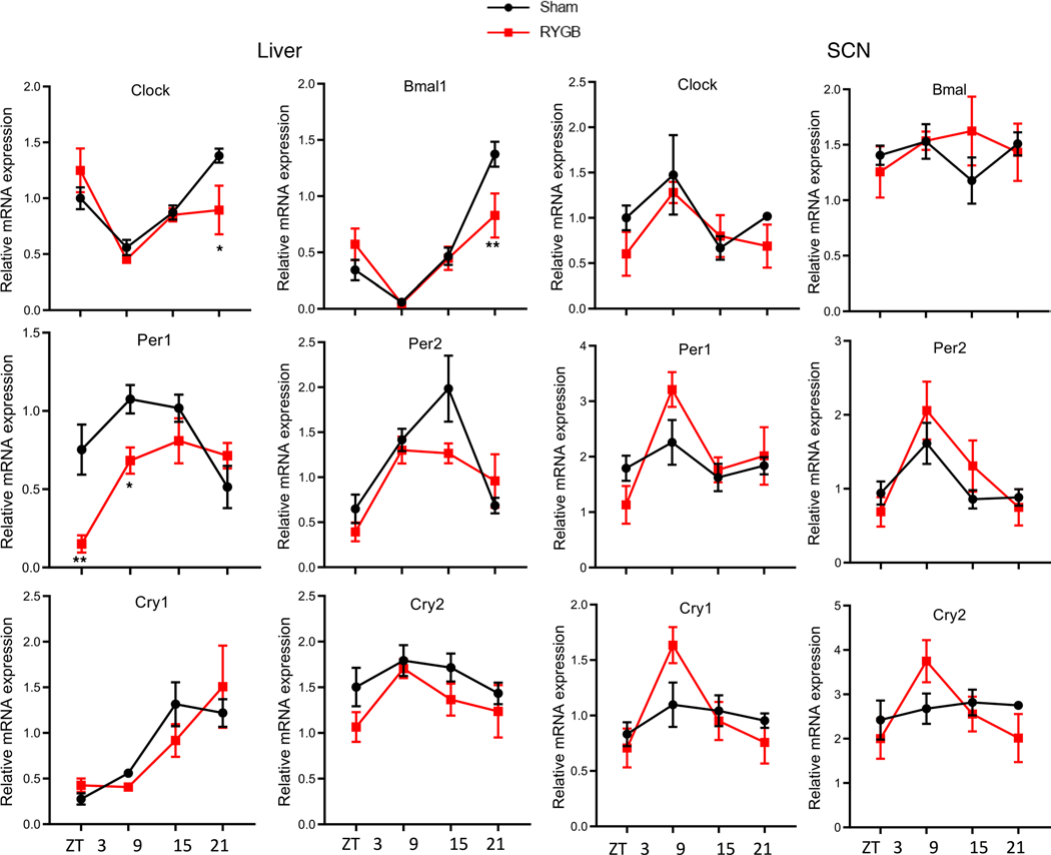
RYGB alters clock gene expression in the liver but not SCN of the hypothalamus. mRNA clock gene expression of *Clock*, *Bmal1*, *Period1* (*Per1*), *Period 2* (*Per2*), *Cryptochrome 1* (*Cry1*), and *Cryptochrome 2* (*Cry2*) in the liver and SCN of RYGB and Sham-operated C57BL/6J mice at variable ZT3, ZT9, ZT15, and ZT21, maintained on HFD and 12-hour light/12-hour dark conditions. Relative gene expression is reported as mean ± SEM. Two-way ANOVA followed by Holm-Šídák multiple comparisons test. *P < 0.05, **P < 0.01. Sham *n* = 6, RYGB *n* = 4–5.

**Figure 4:**
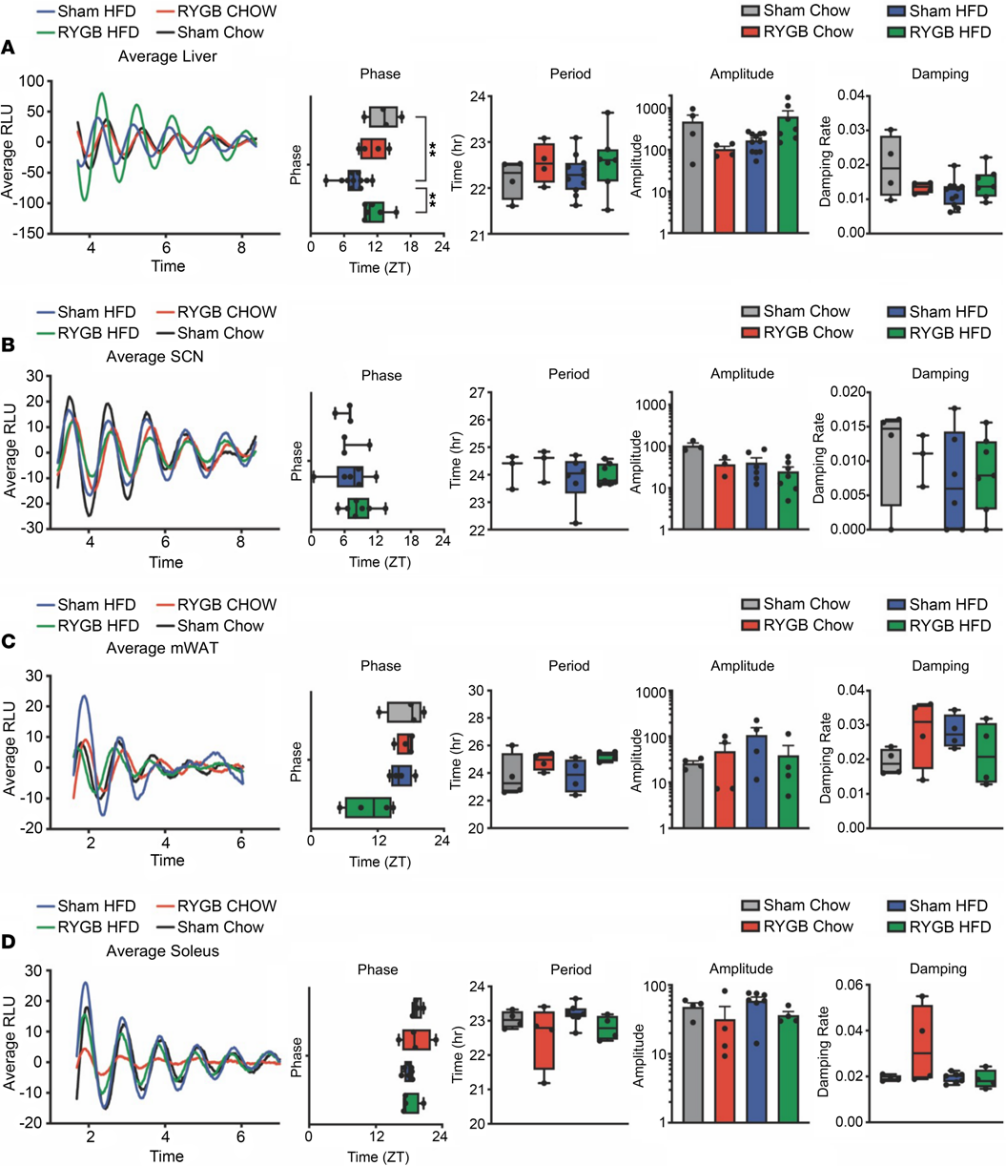
RYGB induces a phase shift in hepatic clock gene oscillation but not the SCN. *mPer2^Luc^* mice were placed on either HFD or regular CHOW diet, and RYGB versus sham surgery was performed on both groups. Tissue analysis was implemented at day 10, when RYGB mice and their sham counterparts had similar body weights. Average background-subtracted bioluminescence showing circadian profiles of mPER2 expression in (**A**) liver, (**B**) SCN, (**C**) mWAT, and (**D**) soleus muscle from *mPer2^Luc^* knockin mice after surgeries. The first 1–3 days of traces were excluded due to nonlinear background. Phase map, period values of mPER2 rhythms, amplitude, and damping were shown for central and peripheral circadian oscillators of *mPer2^Luc^* knockin mice in RYGB and Sham-operated mice on CHOW and HFD. Values reported as means ± SD. Box plots show the interquartile range (box), median (line), and minimum and maximum (whiskers). One-way ANOVA was used to compare 4 groups together. **P < 0.01. *N*, Sham Chow *n*= 3–4, RYGB Chow = 3–4, Sham HFD = 4–6, RYGB HFD = 4–6.

To determine the effect of gastric bypass on the molecular clock per se, we measured mRNA expression of *Clock*, *Bmal1*, *Period 1* (*Per1*), *Period 2* (*Per2*), *Cryptochrome 1* (*Cry1*), and *Cryptochrome 2* (*Cry2*) at 4 time points (or ZTs) across the sleep/wake cycle, in RYGB and sham-operated DIO C57BL/6J mice. *Clock*, *Bmal1*, *Per1*, and *Per2* mRNA expression was significantly altered within the livers of RYGB-operated mice compared with their sham counterparts. On the other hand, there was no significant difference in mRNA expression of any of the clock genes within the SCN between RYGB and sham mice (Figure 3). To examine the effect of gastric bypass on the cell-autonomous circadian rhythms in isolated tissues, we performed RYGB in a *Per2* reporter mouse (*mPer2^Luc^*) (22) model fed either HFD or regular chow (CHOW) before assessing PER2 protein fused to luciferase (PER2:LUC) oscillations in several tissues on postoperative day 10, when RYGB and sham mice had similar body weights (Supplemental Figure 2A). A phase shift in hepatic clock gene (represented by *Per2*) was found in RYGB mice on either diet (HFD or CHOW). In the SCN, however, no changes were detected in the oscillation phase, period, amplitude, or damping, based on PER2:LUC rhythms. Although not statistically significant, mesenteric WAT (mWAT) showed a tendency toward phase advancement, while both mWAT and soleus muscle tissues exhibited a tendency for an attenuated period and wave amplitude post-RYGB that was diet independent (Figure 4).

### RYGB-induced effects on energy balance are significantly attenuated in Clock^Δ19^ mice.

**Figure 5:**
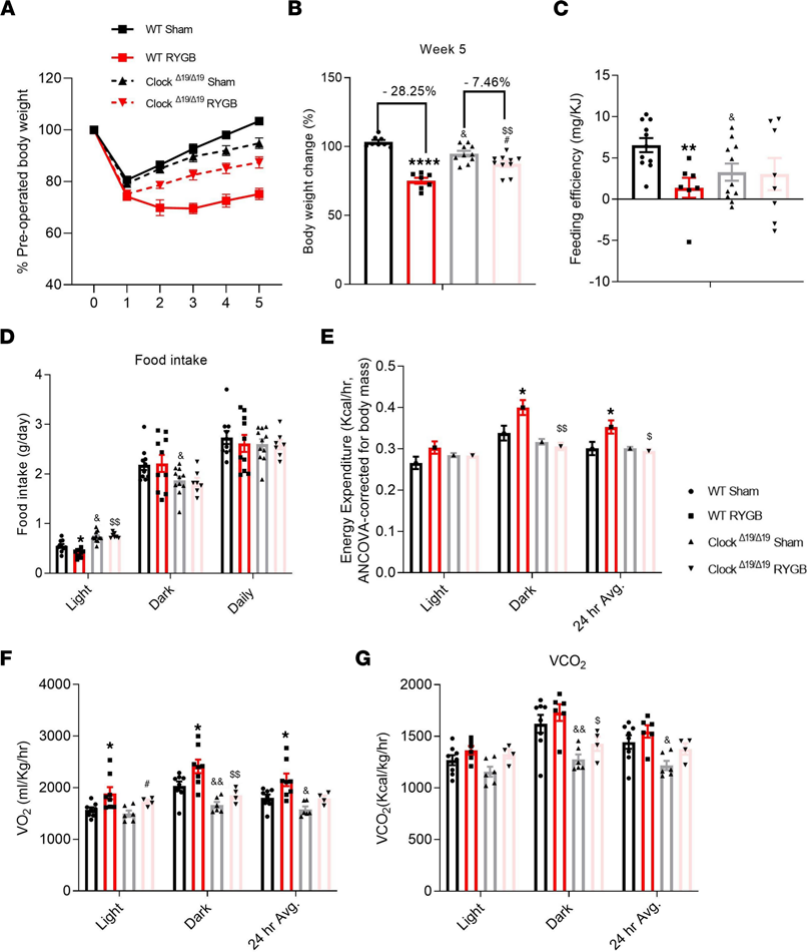
RYGB-induced metabolic effects are significantly attenuated in *Clock*^Δ19^ mice. (**A**) Average weekly body weight expressed in percentage of preoperative weight in RYGB-operated DIO *Clock*^Δ*19/*Δ19^ mice and WT littermates maintained on a 60% HFD diet and under normal light/dark conditions, compared with their sham counterparts. (**B**) Body weight change (%) on week 5 after RYGB, (**C**) feeding efficiency (expressed as mg of body weight gain per KJ consumed), and (**D**) food intake separated in diurnal rhythm during postsurgery day 7 through day 14. (**E**) Total energy expenditure (expressed in kcal/h), measured by respirometry or indirect calorimetry 3 weeks after surgery, using weight-adjusted ANCOVA. (**F**) O_2_ consumption and (**G**) CO_2_ consumption were obtained from measurements taken in the CLAMS using respirometry or indirect calorimetry in free moving animals during postsurgery week 3. Mean ± SEM. WT Sham *n* = 8–11, WT RYGB *n* = 7–10, *Clock*^Δ*19/*Δ19^ Sham *n* = 11, *Clock*^Δ*19/*Δ19^ RYGB *n* = 7–10 (CLAMS data *n* = 4). One-way ANOVA followed by Tukey’s test. *^,^^#,^^$^*P* < 0.05, ^$$,^^&&^*P* < 0.01, ****P < 0.0001. *WT Sham versus WT RYGB, ^#^*Clock*^Δ*19/*Δ19^ Sham versus *Clock*^Δ*19/*Δ19^ RYGB, ^&^WT Sham versus *Clock*^Δ*19/*Δ19^ Sham, ^$^WT RYGB versus *Clock*^Δ*19/*Δ19^ RYGB.

Next, we investigated the requirement of an intact molecular clock machinery for gastric bypass–induced metabolic effects by performing RYGB in DIO *Clock*^Δ19^ mutant mice and WT littermates. As expected and previously reported (23), the *Clock*^Δ19^ mice gained more weight on HFD than their WT littermates (Supplemental Figure 2B). Nonetheless, the effect of RYGB on weight loss was significantly attenuated in *Clock*^Δ19^ mutant mice (28% in WT versus 7.5% in *Clock*^Δ19^) (Figure 5, A and B). There was also a loss of early changes in diurnal eating behavior as well as an increase in energy expenditure that is typically observed post-RYGB in WT DIO mice (Figure 5, C–G). In addition, even though RYGB had no effect on total locomotor activity during either light or dark cycle, *Clock*^Δ19^ mutant mice displayed a significant reduction in spontaneous locomotor activity during the dark cycle, compared with their WT littermates (Supplemental Figure 3A). Furthermore, we found that the initial reduction in the light cycle post-RYGB (Figure 5D) was mainly due to reduction in meal size and meal frequency (Supplemental Figure 3, B and C), a phenomenon that was absent in the *Clock*-deficient mice.

### Clock is required for RYGB-induced glucoregulatory changes.

**Figure 6:**
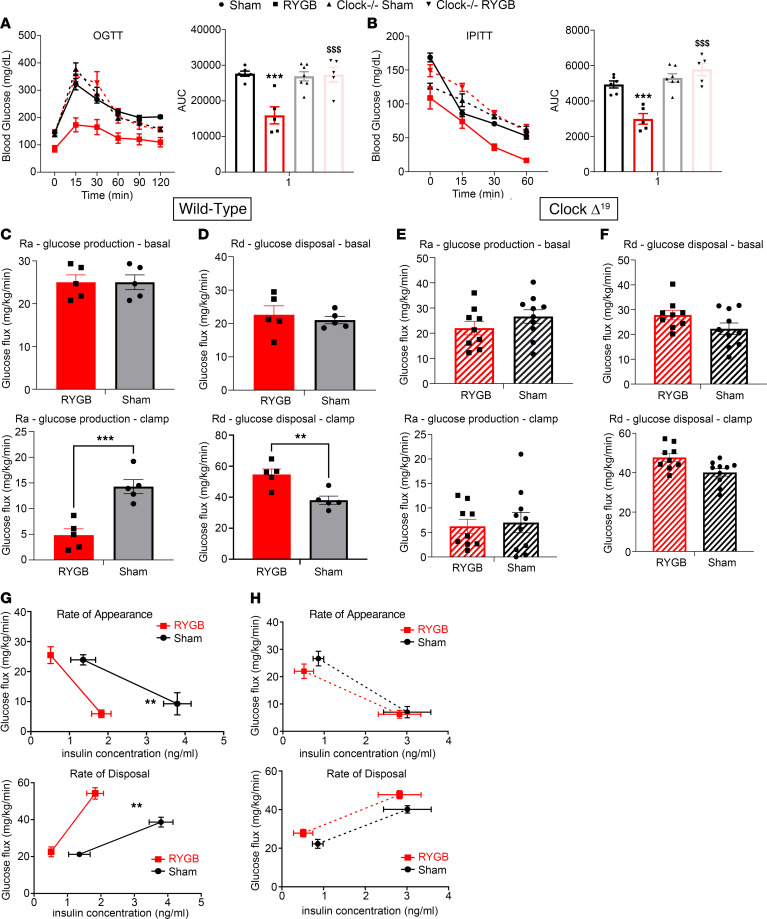
*Clock* is required for RYGB-induced glucoregulatory changes (week 6 postsurgery). (**A**) Oral glucose tolerance test and (**B**) insulin tolerance test performed during postoperative week 4 or 5 in RYGB-operated DIO *Clock*^Δ*19/*Δ19^ mice and WT littermates compared with their sham counterparts. Mean ± SEM. WT Sham *n* = 6, WT RYGB *n* = 5, *Clock*^Δ*19/*Δ19^ Sham *n* = 7, *Clock*^Δ*19/*Δ19^ RYGB *n* = 5. One-way ANOVA followed by Tukey’s test. ***^,^^$$$^*P* < 0.001. *WT Sham versus WT RYGB, ^$^WT RYGB versus *Clock*^Δ*19/*Δ19^ RYGB. (**C**–**H**) Hyperinsulinemic-euglycemic clamps were performed during week 6 in RYGB DIO *Clock*^Δ*19/*Δ19^ and WT littermates compared with their sham counterparts. (**C** and **D**) Basal and clamped glucose production (Ra) and glucose disposal rate (Rd) in WT RYGB versus sham-operated mice. (**E** and **F**) Basal and clamped glucose production and glucose disposal rate in *Clock*^Δ*19/*Δ19^ RYGB versus sham-operated mice. (**G** and **H**) Glucose production and glucose disposal rate versus insulin concentration (in ng/mL) in WT and *Clock*^Δ*19/*Δ19^ RYGB mice and their sham counterparts. Mean ± SEM. WT Sham *n* = 11, WT RYGB *n* = 10, *Clock*^Δ*19/*Δ19^ Sham *n* = 11, *Clock*^Δ*19/*Δ19^ RYGB *n* = 10. One-way ANOVA followed by Tukey’s test. **P < 0.01, ***P < 0.001. WT Sham versus WT RYGB.

**Figure 7:**
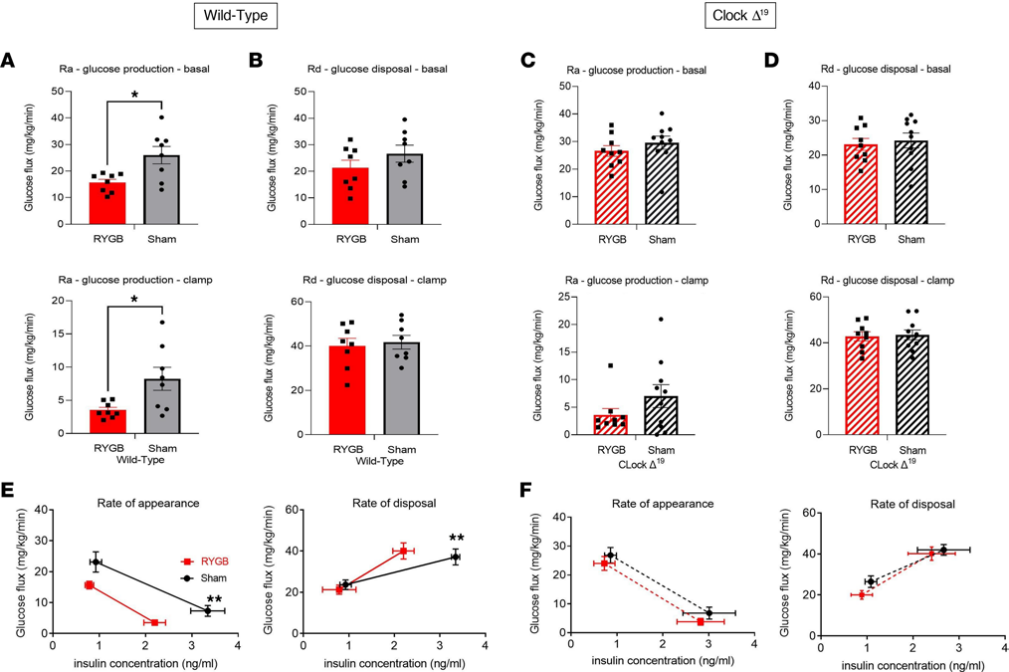
RYGB fails to improve early hepatic glucose production in DIO *Clock^Δ19^* mutant males (week 2 postsurgery). Hyperinsulinemic-euglycemic clamps were performed during week 2 in RYGB-operated DIO *Clock*^Δ*19/*Δ19^ and WT littermates compared to their sham counterparts. (**A** and **B**) Basal and clamped glucose production (Ra) and glucose disposal rate (Rd) in WT RYGB versus sham-operated mice. (**C** and **D**) Basal and clamped glucose production and glucose disposal rate in *Clock*^Δ*19/*Δ19^ RYGB versus sham-operated mice. (**E** and **F**) Glucose production and glucose disposal rate versus insulin concentration (in ng/mL) in WT and *Clock*^Δ*19/*Δ19^ RYGB-operated mice and their sham counterparts. Mean ± SEM. WT Sham *n* = 9, WT RYGB *n* = 8, *Clock*^Δ*19/*Δ19^ Sham *n* = 8, *Clock*^Δ*19/*Δ19^ RYGB *n* = 8. One-way ANOVA followed by Tukey’s test. *P < 0.05. WT sham versus WT RYGB.

*Clock*^Δ19^ mice failed to show improvement in glucose tolerance or insulin sensitivity post-RYGB, like what was observed in WT littermates (Figure 6, A and B). The glucose homeostatic findings were further investigated using a hyperinsulinemic-euglycemic clamp. RYGB reduced endogenous glucose production and increased glucose disposal in the clamped (i.e., hyperinsulinemic) state. Interestingly, such effects were not present in the *Clock*^Δ19^ mice (Figure 6, C–F). Similarly, serum insulin levels were reduced post-RYGB during both basal and clamped conditions in WT but not *Clock*^Δ19^ mice (Figure 6, G and H). Furthermore, when tissue-specific glucose disposal post-RYGB was examined using a radiolabeled tracer, mWAT showed an increase in 2-deoxyglucose (2-DG) uptake in WT but not *Clock*^Δ19^ mice (Supplemental Figure 4). Finally, we examined whether the early change in circadian eating behavior and its associated improvement in glucose tolerance test results after gastric bypass (Figure 2A) is associated with an alteration in hepatic glucose flux, independent of weight change, and whether the molecular clock is necessary for this early RYGB-induced effect. We performed a hyperinsulinemic-euglycemic clamp in *Clock*^Δ19^ mice and WT littermates 2 weeks post-RYGB to study glucose flux in a weight-independent manner. As expected, *Clock*^Δ19^ exhibited no significant early changes in glucose flux compared with WT littermates, which displayed improvement in basal (fasting) and clamped hepatic glucose production during the same period (Figure 7).

## Discussion

In this study, we show that RYGB reverses the HFD-induced disruption in the diurnal feeding rhythm and induces a phase advance in hepatic clock genes that is weight independent. In addition, we demonstrate that this early change in energy consumption behavior is associated with improved oral glucose tolerance in the rest/fasting phase (ZT2). This latter finding was also weight independent, as the PF mice were similar in body weight to their bypass counterparts. Interestingly, RYGB mice exhibited higher peripheral insulin sensitivity than both PF and ad libitum–fed sham mice, suggesting that RYGB regulates glucose homeostasis at multiple levels. Furthermore, we did not find evidence that RYGB affects central clock expression or its ex vivo oscillation. Notably, even though RYGB induced some weight loss in the *Clock*^Δ19^ mutant mouse, we find that the glucoregulatory effects of RYGB that were previously reported to be weight independent in rodents (14, 15) are completely lost in the *Clock*^Δ19^ mice. We further reveal that these early glucose homeostatic improvements seen post-RYGB reflect a decrease in endogenous hepatic production, again an effect that is lost in the *Clock*^Δ19^ mutants.

Bariatric surgery has been available for decades and has been recognized as a foundation in the treatment of morbidly obese patients with type 2 diabetes (24). However, an understanding of the mechanisms through which RYGB regulates glucose metabolism and other obesity-associated diseases could allow for development of less invasive targeted therapies. Two previous reports found no major effect of VSG, another common bariatric procedure, on circadian molecular rhythms or clock gene expression. Furthermore, the main energy and glucoregulatory effects of VSG were preserved in *Clock*^Δ19^ mutant mice, suggesting that the molecular clock is not involved (or at least not required) for metabolic regulation post-VSG (20, 21). On the other hand, VSG did alter transcriptional rhythms regulating lipid metabolism within WAT (21). Despite their previously reported humoral similarities (25), RYGB and VSG have recently been recognized to exhibit several differences in their metabolic effects, at least in rodents (19, 26). Here we show that RYGB also diverges from VSG in its effect on circadian feeding behavior and molecular clock expression (specifically within the liver), which is associated with early reduction in hepatic glucose flux. The liver clock is the most sensitive of all peripheral tissues to entrainment by feeding cycles (5, 6), and it can also synchronize all other peripheral tissues to the eating behavior (11), while its rhythm is also responsive to insulin (27). Our present findings indicate that RYGB induces a transient early change in the diurnal eating rhythm, a change that is closely associated with changes in the phase shift in the hepatic clock and subsequently early reduction in hepatic glucose production. Remarkably, we also show that RYGB-operated animals that are on HFD synchronize their hepatic “clock” oscillation to a pattern similar to animals of regular CHOW diet (either sham or RYGB operated). This effect, interestingly, was weight independent (Supplemental Figure 2). It has been suggested that the homeostatic glucose regulation following gastric bypass occurs at multiple levels, such as through increased secretion of incretins (28), improved pancreatic β cell function and insulin secretion (29), as well as enhanced overall insulin sensitivity (30).

Our present findings indicate that one mechanism for the beneficial metabolic effects of RYGB may be to maintain the phase of the liver oscillator in synchrony with other oscillators. A previous rodent study showed that when rats were provided with a choice of chow versus HFD, RYGB-operated rats consumed more chow than HFD but fewer total calories overall during the dark cycle (31). Remarkably, a small human study using hyperinsulinemic-euglycemic clamp early and late post-RYGB showed an early improvement in hepatic glucose secretion and hepatic insulin sensitivity 1 month after surgery, which is later followed by a more pronounced effect on glucose disposal (i.e., peripheral insulin sensitivity) (32). It is well recognized that humans with disrupted circadian rhythm, such as shift workers, are predisposed to obesity, type 2 diabetes, and metabolic syndrome, and they appear to have an attenuated weight loss response to bariatric surgery when compared with controls (33, 34). We speculate that the early changes in hepatic glucose homeostasis that are associated with the diurnal eating behavior are likely due to the anatomical rearrangements of the surgery, which include changes in microbiome and bile acid flux and subsequent alteration in energy signals along the gut/brain or gut/liver axis. These changes are likely responsible for reprogramming of the peripheral hepatic clock, which is tightly associated with the alteration in diurnal feeding pattern. Moreover, we recently showed that diet-induced thermogenesis within WAT is tied to and dependent on adipocytes’ *Clock* gene expression (35). This is highly supportive of our findings that RYGB failed to induce an increase in energy expenditure in the *Clock*^Δ19^ mutant mice, a phenotype that was not present in the VSG model (20). Although our study disclosed that global functional deficiency of the molecular clock machinery significantly attenuates the RYGB-evoked energy and glucoregulatory changes, we have yet to determine if the hepatic clock alone (or in synchrony with the central/ hypothalamic clock) is necessary for the glucose homeostatic changes postsurgery. Our recent published data showing that a functional circadian clock within visceral WAT regulates time-restricted diet-induced thermogenesis (35) combined with our attenuated energy expenditure data in *Clock*^Δ19^ mice post-RYGB suggest that adipose tissue’s clock may be necessary for RYGB-induced energy balance. Finally, plenty of data in the literature, including a recent study, show that early-onset caloric restriction has a positive effect on longevity in male mice (36). Large human cohorts and retrospective study analysis suggest that bariatric surgery decreases not only cancer and cardiovascular disease–related mortality but also all-cause mortality (37–39). Our study did not examine the effect of RYGB on longevity in this DIO mouse model, and despite RYGB-operated mice not maintaining a phenotype of reduced energy consumption as in humans, it would be interesting to examine the implications of this transient feeding restriction postsurgery on mice’s longevity in future studies.

On the other hand, we understand that the *Clock*^Δ19^ mouse model that we used in our experiments already expresses a metabolically abnormal phenotype of obesity, hyperglycemia, and lipidemia. However, other models of clock deficiency, such as *Bmal1*-knockout mice, are prone to obesity, which is exacerbated by HFD, but they are protected from hyperglycemia, lipidemia, and fatty liver and fibrosis (39). Using such a model would create a selection bias of an animal that does express the manifestation of hepatic steatosis, hyperglycemia, or hyperlipidemia that exist in the DIO animal model mimicking the features of metabolic syndrome that is observed in obese humans.

In summary, our data indicate that a normalized (time-restricted) feeding rhythm is a key factor in RYGB-induced early glucoregulatory response. Therefore, it may be important to monitor meal timing and other obesity-promoting abnormal circadian behaviors (such as frequent or late-night snacking) in patients with type 2 diabetes to ensure the best response to gastric bypass. Furthermore, the difference in response between VSG and RYGB in a model of a “broken” circadian rhythm (or deficient clock machinery) could represent an opportunity for health care providers managing obesity to select or avoid certain surgeries in patients with altered circadian behavior.

## Methods

### RYGB surgeries of mice

All animals were given a weight-based dose of antibiotics (enrofloxacin) as prophylaxis for perioperative infection and analgesics (buprenorphine) for pain management before laparotomy. The surgical rearrangement of Roux-en-Y anatomy and the sham control were performed as described in our previous studies (19). After removing hair with an electric razor, the abdomen skin was sterilized by 3 alternating scrubbings with betadine and 70% alcohol, then wiped with a betadine prep pad. Midline laparotomy was performed using forceps and scissors and extended from the xiphoid to umbilicus. The abdominal wall was entered using forceps and sterile cotton swabs. The distal transected jejunal limb is connected to the proximal anterior gastric wall and the proximal transected jejunal limb to the afferent Roux limb. The proximal gut was excluded from alimentary flow using a hemostasis clip (Ethicon Endosurgery) placed immediately distal to the gastro-jejunostomy. Closure was performed using 4−0 Vicryl (or PDS) with a running suture in the abdominal wall and interrupted sutures using 4−0 nylon + Vetbond Tissue adhesive glue in the skin. The sham procedure involved gastrotomy, enterotomy, and repair. All mice recovered from the stress of surgery in their home cages, with prophylactic injection of analgesics daily for the first 2 days and then on an as-needed basis. Mice were placed on liquid diet and supplemental intraperitoneal injections of dextrose fluid for hydration and nutrition during postoperative week 1. They were all allowed to recover on solid diet during postoperative week 2. Body weight was measured weekly.

### Study design

Several groups of 15- to 16-week-old DIO C57BL/6J mice with an appropriate body weight (40–45 g) underwent RYGB versus sham surgery. Experiments were performed between postoperative weeks 1 and 6. Age-matched lean C57BL/6J males were used as controls. One group of mice was dedicated for 24-hour (12 hours/12 hours) diurnal food intake recordings for 5 weeks. A second group included a pair-feeding experiment where a group of DIO shams was PF day by day to the RYGB group, but food was provided only during the dark cycle. Weight change was tracked over 4 weeks. Glucose tolerance tests (GTTs) were performed twice between weeks 1 and 2 during 2 ZTs. The first GTT was performed during the early light cycle (fasting phase), ZT2, and the second during the early dark cycle (feeding phase), ZT14. The 2 GTTs were separated by 72 hours. Similarly, ITTs were performed twice between week 1 and 2, at ZT2 and ZT14, 72 hours apart. Finally, a third large group of DIO C57BL/6J was generated to examine molecular clock gene expression in multiple tissues. Mice were sacrificed at ZT3, ZT9, ZT15, and ZT21 (6 weeks postintervention). mRNA expression of *Clock*, *Bmal1*, *Per1*, *Per2*, *Cry1*, and *Cry2* genes was assessed by real-time quantitative PCR (see Supplemental Methods). Another group of DIO *Clock*^Δ*19/*Δ19^ mice and their WT littermates were dedicated for measurement of energetics, body composition, and total energy expenditure using the CLAMS (metabolic cage system). Finally, 2 separate groups were generated for euglycemic-hyperinsulinemic clamping, to study the early effect of RYGB, at both week 2 and week 6 postsurgery. Two groups of *mPer2^Luc^* knockin mice were placed on HFD until they reached a weight appropriate for surgery, at which time they underwent either RYGB or sham procedure as described above. At day 5 postoperatively, one group was placed back on HFD and the other on chow diet for another 5 days. Both groups were sacrificed at day 10, and tissues were collected for clock gene expression analysis.

### Diurnal energy consumption measurement

Diurnal energy consumption was measured in RYGB, sham, and lean groups from postoperative day 8 to 35. Measurements of HFD or regular chow were recorded at ZT0 (6 am) and ZT12 (6 pm). Food intake of HFD and regular chow was multiplied by 5.21 and 3.82, respectively, to convert mass (g) to kilocalories.

### Pair-feeding experiment

At the end of week 1 postsurgery, one cohort of DIO (HFD-fed) shams were PF to a group of RYGB-operated mice on a day-to-day basis while providing food only at the beginning of the dark cycle (6 pm) and removing any possible remaining food at the beginning of the light cycle (6 am). This group was called dark-cycle PF shams. Another group of ad libitum–fed shams were kept as control, also on HFD, in addition to the RYGB group. All 3 groups were followed for 4 weeks after surgery with weekly body weights recorded. Two separate GTTs and ITTs were performed on all 3 groups between weeks 1 and 2 postsurgery at ZT2 (8 am) and ZT14 (8 pm).

### GTT

Following a 12-hour (ZT2) or 2-hour fast (ZT14) during postsurgery weeks 1–2, mice were administered 1 g/kg body weight of d-glucose (50-99-7, RPI) by oral gavage. Blood glucose was measured from tail vein blood using a handheld glucometer (Contour, Bayer HealthCare LLC), immediately before and 15, 30, 60, 90, and 120 minutes after glucose administration.

### ITT

During postoperative weeks 1–2, mice were administered 0.75 U/kg body weight dosage of insulin (NDC 0002-8215-01, HI-210, Humulin R, Eli Lilly & Co) by intraperitoneal injection. Tests were performed twice, at ZT2 and ZT14. Blood glucose was measured from tail vein blood using a handheld glucometer, described above, immediately before and 15, 30, 60, 90, and 120 minutes after insulin administration.

### Circadian activity analysis in mPer2Luc knockin mice

Mice that expressed PER2:LUC were euthanized. SCN, liver, soleus, and WAT tissues were excised. Tissues were placed in a LumiCycle (Actimetrics), at 37°C, as previously described (22). For SCN, the brain was placed in a vibrotome (Leica VT1200 S), then cut into 300 μm–thick coronal slices around the SCN region. The SCN was further isolated using a sterile scalpel. The full-length soleus muscle was isolated, whereas anatomically consistent sections of the liver and mWAT were used. All isolated tissues were spread flat on a thin filter (0.2 μm, MilliporeSigma), which on its basal side was bathed in 1.2 mL of luciferin-containing media (DMEM, Gibco). The medium contained sodium bicarbonate (352.5 μg/mL), 10 mM HEPES (Gibco), 2 mM l-glutamine, 2% B-27 serum-free supplement (Invitrogen), penicillin (25 U/mL), streptomycin (Gibco, 20 μg/mL), as well as 0.1 mM luciferin sodium salt (Biosynth AG). A round coverslip was placed on top of the dishes, which were sealed with vacuum grease. Background-subtracted data were fit to a cosine curve of equation:
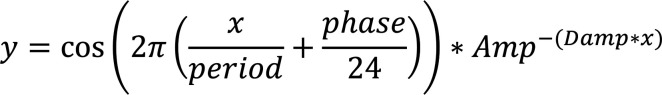


using sum of least squares fit to quantify phase, amplitude, period, and damping rate.

### Measurement of total energy expenditure using indirect calorimetry

Feeding pattern, locomotor activity, and energy expenditure were all determined between postoperative weeks 2 and 3, using the CLAMS at the University of Iowa Metabolic Phenotyping Core Facility. For acclimatization, mice were individually housed for 5 days. Mice were then moved into electronically monitored cages, with 24-hour access to food and water. Data of O_2_ consumption, CO_2_ production, and locomotor activity were continuously recorded 24 hours per day, with daily welfare check of mice.

### Feeding efficiency calculation

Feeding efficiency was calculated as the rate of body mass gain per amount (mass, or caloric content) of food ingested by a mouse.

### Hyperinsulinemic-euglycemic clamp

Two groups of RYGB and sham-operated mice from both genotypes (WT and *Clock*^Δ*19/*Δ19^) were transferred to the Metabolic Phenotyping Core Facility, where a vascular catheter for clamping was placed under anesthesia in the jugular vein. One group underwent catheter insertion at week 2 postsurgery and the other group at week 6. A high, normal, or low-dose euglycemic-hyperinsulinemic clamp was performed in the postabsorptive state (i.e., ~5 hours fasting) in conscious mice as follows.

#### Catheter placement.

A sterile silicone catheter (0.012′′ inner diameter, Liveo, DuPont, catalog 1118914) was placed into the right jugular vein of mice under isoflurane (Akorn Animal Health catalog 140109) anesthesia. The catheter was flushed with 200 U/mL heparin (catalog 63739-931-14; McKesson Corp) in saline (Baxter catalog 992 167). The free end of the catheter was directed subcutaneously and connected to a small tubing device (in-house preparation) that exits through the back of the animal. Mice received postoperative care in accordance with IACUC guidelines. Mice were allowed to recover from catheter placement surgery for 6–7 days before undergoing hyperinsulinemic-euglycemic clamps. Animals showing impaired recovery (as evidenced by wound infection, weight loss over 10% compared to presurgery weight) were excluded from further experiments.

#### Glucose and insulin measurement experiments.

Hyperinsulinemic-euglycemic clamps were performed on unrestrained, conscious mice. Micro-renathane tubing (0.033′′ inner diameter, Braintree catalog MRE033) was used to connect infusion pumps (Harvard Apparatus, catalog 704501) to the infusion catheter via a swivel (Instech, catalog 375/D/22QM) that allows free movement of the mouse. Mice were fasted for 5 hours prior to starting insulin and glucose infusion (time 0). d-[3-3H]-glucose (PerkinElmer, catalog NET331C001MC, 20 Ci/mMol — reconstituted in saline to obtain final concentration of 0.1 μCi/μL) was employed as a glucose turnover tracer. For the basal period (time –80 to time 0), following a bolus infusion of 1 μCi in 1 minute, a steady 0.1 μCi/min infusion rate was used. Blood samples (~20–25 μL) from tail cut were collected using heparinized glass capillary tubes (Thermo Fisher Scientific, catalog 22-362566) at times –80, –20, –10, and 0, then centrifuged at 10,000*g* for 5 minutes at 4°C, and plasma was transferred to a fresh tube and stored frozen at –80°C until use. For the pre-clamp and clamp periods (i.e., from time 0 until the end of experiment) d-glucose was infused at the rate 0.2 μCi/min (2 μL/min). Also, at time 0, insulin (Humulin, Eli Lilly & Co, catalog NDC 0002-8215-01) administration at the dose of 4 mU/min/kg body weight was initiated (using a separate infusion pump) with a 1-minute, 10 μL bolus, followed by continuous infusion at 1 μL/min throughout pre-clamp and clamp periods. Simultaneously, infusion of 50% dextrose in saline (Hospira, Inc, catalog NDC 0409-66-02) was started with variable infusion rates aimed at reaching and maintaining the blood glucose level at 150 mg/dL. Blood glucose levels were measured using a glucometer every 10 minutes, and the glucose infusion rates were adjusted accordingly. Once the desired blood glucose level was stable (typically after 70–110 minutes), a glucose uptake tracer (2-DG [14C], PerkinElmer, catalog NEC495001MC, 56 mCi/mMol reconstituted in saline) was administered as a single bolus at a dose of 13 μCi in 96 μL of saline over 1 minute at time 75. Blood samples (~20–25 μL) from tail cut were collected at times 80, 90, 100, 110, and 120 minutes, then centrifuged at 10,000*g* for 5 minutes at 4°C, and plasma was transferred to a fresh tube and stored frozen at –80°C until use. After the clamp period, mice were anesthetized with isoflurane, and tissues of interest were harvested and frozen. Typically, it took 2–3 minutes to anesthetize a mouse and around 2–3 minutes to harvest tissues (time depends on number of tissues needed). Glucose concentrations in the plasma and in the 50% dextrose infusates were measured using an enzymatic method (Analox Instruments Ltd, catalog GMD9). Plasma samples (5 μL) were diluted with 10 μL of saline, treated with 70 μL of 0.1N Ba(OH)_2_ and 70 μL of 0.1N ZnSO_4_ (both from MilliporeSigma), then centrifuged at 16,000*g* for 5 minutes at 4°C. Supernatant (80 μL) was collected and dried overnight at 50°C, then reconstituted in 200 μL of distilled water. Chemical Standard-Evaporated and Chemical Recovery Standard standards were determined by treatment of diluted (1:200 in water) d-[3-3H]-glucose infusate in the same manner as plasma samples. Tracer (3H and 14C) activity was measured using liquid scintillation counter (PerkinElmer). The obtained values were used for determination of the relevant glucose turnover parameters (Rd and Ra). We adjusted for small deviations from steady state by use of Steele’s equations (40). A smaller (typically 20–40 mg) tissue sample was then obtained and placed in 750 μL of 0.5% perchloric acid, homogenized using a mechanical device (TissueLyzer, QIAGEN), and then centrifuged at 4,000*g* for 20 minutes to remove insoluble residue. Supernatant (625 μL) was collected, neutralized with 30 μL of 2 M KOH, then centrifuged at 10,000*g* for 10 minutes. All procedures were performed at 4°C. Supernatant (62.5 μL) was used for measuring total tracer (14C) activity using a liquid scintillation counter.

### Animals and protocols

Lean WT C57BL/6J and Clock^m1Jt^/J (*Clock*^Δ19^) mice on C57BL/6J background were purchased from The Jackson Laboratory (strain number 002923). Heterozygous *Clock*^Δ19^ (*Clock*^Δ*19/*+^) male and female mice were bred to produce homozygous *Clock*^Δ19^ (*Clock*^Δ*19/*Δ19^), heterozygous *Clock*^Δ19/+^, and WT (*Clock^+/+^*) mice. Mice were maintained on normal chow diet (Teklad Diets 7913 NIH 31) after weaning until they reached 6 weeks of age. Several groups of mice were switched to an HFD (Research Diets, D12492) to induce obesity as described below. All mice were maintained on a 12-hour light/12-hour dark cycle. Water and food were available ad libitum except when mice were fasted as described below. The *mPer2^Luc^* knockin mice, obtained from Northwestern University, have luciferase fused to the *Per2* gene as a real-time reporter of clock gene circadian dynamics (21). They are on a C57BL/6J background, are bred as heterozygous males and females at Northwestern University, and were housed under similar experimental conditions. All the *mPer2^Luc^* knockin experiments were performed in the lab at Northwestern University.

### Statistics

Data are expressed as mean ± SEM and analyzed by 1- or 2-way ANOVA, followed by Tukey-Kramer post hoc analysis, using Prism 9.0 (GraphPad Software). A *P* < 0.05 was considered statistically significant. ANCOVA was used to correct energy expenditure data for body mass using SPSS, as indicated by the term “ANCOVA-corrected for body mass.” Extreme outliers were removed from data set prior to analysis when meeting the criteria of being above or below 3 SDs of the corrected mean value.

### Study approval

Care of all animals and procedures were approved by the Animal Care and Use Committees at the University of Iowa and Northwestern University.

## Author contributions

MM, JB, and YY conceived the idea and planned the experiments. MM, YY, MAEH, RO, HH, LT, BL, YC, DFG, DCL, and JC collected and analyzed data. MM and YY wrote the manuscript. JB, KR, and MM obtained funding.

## Supplementary Material

Supplemental data

## Version 1 02/14/2023

In-Press Preview

## Version 2 03/22/2023

Electronic publication
