# Supplementary material for: Gastric bypass alters diurnal feeding behavior and reprograms the hepatic clock to regulate endogenous glucose flux
Source: JCI Insight. 2023 Mar 22;8(6):e166618. doi: 10.1172/jci.insight.166618 (PMC10070113; doi:10.1172/jci.insight.166618)
Supplement: Supplemental data [file jciinsight-8-166618-s128.pdf]

Gastric bypass alters diurnal feeding behavior and reprograms hepatic clock to regulate endogenous glucose flux

Yuanchao Ye[1], Marwa Abu El-Haija[2,3], Reine Obeid[4], Hussein Herz[1], Liping Tian[5], Benjamin Linden[1], Yi Chu[1], Deng Fu Guo[6,10], Daniel C. Levine[7$], Jonathan Cedernaes[7], Kamal Rahmouni[1,6,8,9,10], Joseph Bass[7], Mohamad Mokadem[1,6,8,9,10*]

Affiliations:
[1]Department of Internal Medicine, University of Iowa Carver College of Medicine, Iowa City, IA, 52242, USA
[2]Stead Family Department of Pediatrics, University of Iowa Carver College of Medicine, Iowa City, IA, 52242, USA
[3]Department of Pediatrics, Division of Gastroenterology, Hepatology, and Nutrition, Stanford University School of Medicine, Palo Alto, CA, 94304, USA
[4]Department of Biology, American University of Beirut, Beirut, 10150, Lebanon
[5]Department of Clinical Pharmacy, School of Basic Medicine and Clinical Pharmacy, China Pharmaceutical University, Nanjing, Jiangsu, PR China, 211198
[6]Department of Neuroscience and Pharmacology, University of Iowa Carver College of Medicine, Iowa City, IA, 52242, USA
[7]Department of Medicine, Feinberg School of Medicine, Northwestern University, Chicago, IL 60611, USA
[8]Fraternal Order of Eagles Diabetes Research Center, University of Iowa, Iowa City, IA, 52242, USA.
[9]Obesity Research & Education Initiative, University of Iowa, Iowa City, IA, 52242, USA.
[10]Veterans Affairs Health Care System, Iowa City, IA, 52242, USA.
[$] Current Affiliation: Department of Neurology, University of California San Francisco, San Francisco, CA, 94143, USA

***Address correspondence to:**
Mohamad Mokadem, MD
University of Iowa
200 Hawkins Drive, 4570 JCP
Iowa City, IA, 52242
Phone: 319-384-6178; Fax: 319-354-7981
E-mail : mohamad-mokadem@uiowa.edu

Supplemental Methods

Real-Time Quantitative PCR

To measure circadian-related gene expression, total RNA was isolated from liver and SCN using Direct-zol™ RNA MiniPrep kit (Zymo Research, Irvine, CA). Total RNA (1 µg) was reverse transcribed to cDNA using cDNA High-Capacity Reverse Transcription Kit (Applied Biosystems, ThermoFisher). Real-time quantitative PCR (10 ng cNDA, 0.5 µM primers) was performed in duplicates using iQ™ SYBR® Green (Bio-Rad, Hercules, CA) per manufacturer's instructions.

The following primers were used:

Clock Forward	AGA CAT CGC TGG CTG TGT TA
Clock Reverse	TCA GAC CCT TCCTCC ACA CC
Bmal Forward	ATC GCA AGA GGA AAG GCA G
Bmal Reverse	GTG GGC CTC CCT TGC ATT
Per1 Forward	GCC TCC TTG CTA CAG GTA CA
Per1 Reverse	GCT GTC CAG GCA GTT GATC
Per2 Forward	AGC TTC ATC AAC CCG TGG AG
Per2 Reverse	GGG ACA GGC TGC ATC AGT AG
Cry1 Forward	CAC TGG TTC CGA AAG GGA C
Cry1 Reverse	AGC AAA AAT CGC CAC CTG TT
Cry2 Forward	CGG GGA CTC TGT CTA TTG GC
Cry2 Reverse	GCA TCT CCG TCA CTC TAG CC
rps18 Forward	CTG CCA TTA AGG GCG TGG
rps18 reverse	TGA TCA CTC GCT CCA CCT CA

Ribosomal related protein s18 (*Rps18*) mRNA expression was used as an internal control to normalize mRNA expression of these genes.

Supplemental Figures

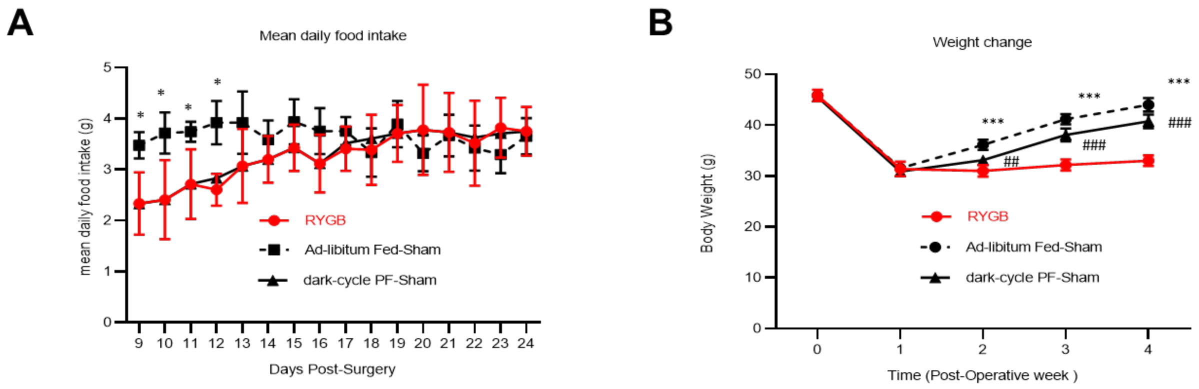

Supplemental Figure 1. (A) Mean daily food intake of HFD in (g) for RYGB, Ad-libitum Fed-Sham and dark-cycle pair fed (PF)-Sham over days post-surgery. (B) Weight change in (g) of RYGB , Ad-libitum Fed-Sham and dark-cycle pair fed (PF)-Sham over time in weeks. Average food intake (at each post-operative day) and body weights at each post-operative week were compared using Student's t-test. $^{***}p<0.001$, $^{*}p<0.05$ for Ad-libitum Fed-Sham vs RYGB, $^{\#\#\#}p<0.001$, $^{\#\#}p<0.01$ for dark-cycle PF-Sham vs RYGB. N, RYGB, n= 7, Ad-libitum Fed-Sham, n = 8, dark-cycle PF-Sham, n =7.

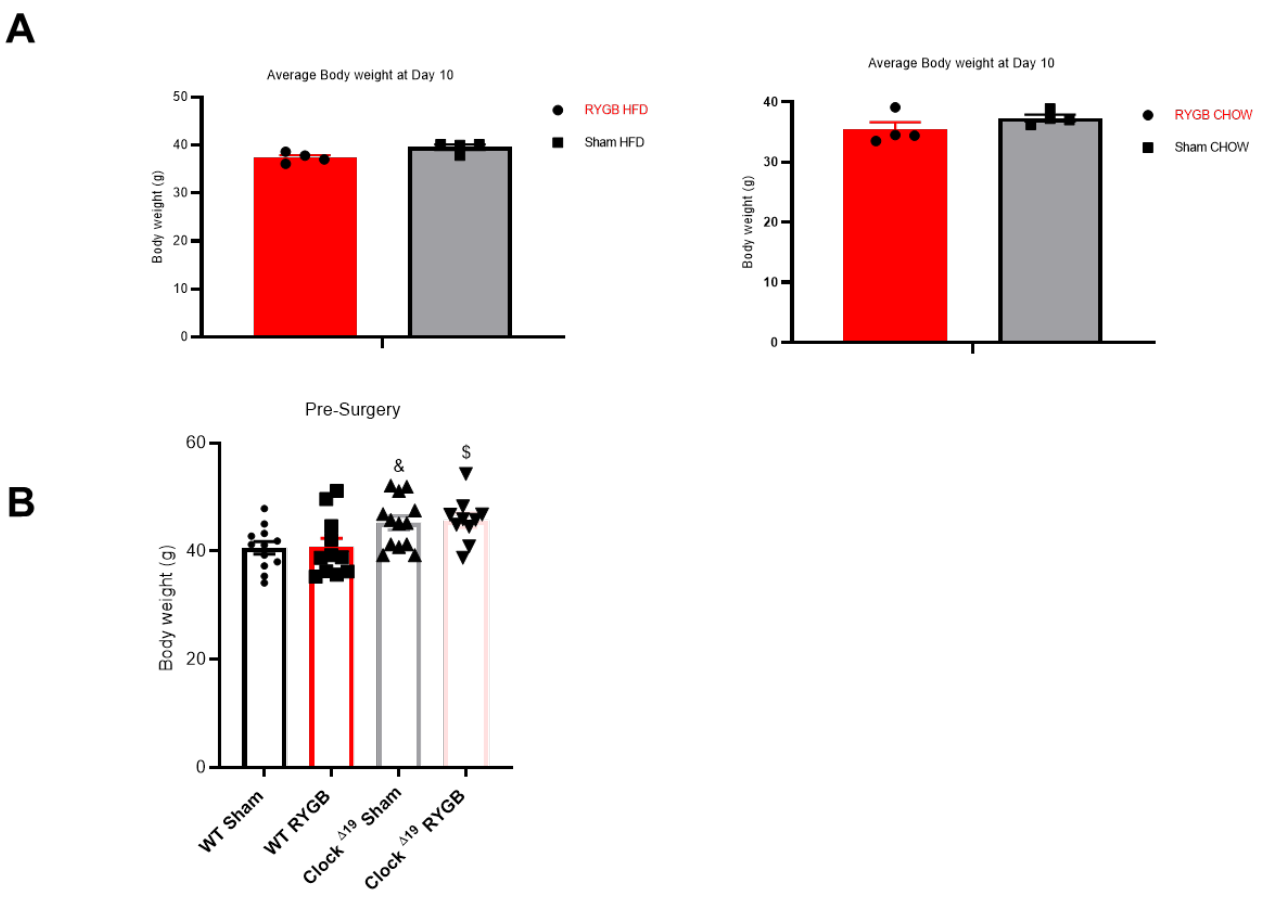

Supplemental Figure 2. (A) Average body weight in (g) post- RYGB in *mPer2*^{Luc} knock in mice at day 10 post-RYGB while on HFD and CHOW diet compared to their Sham counterparts. Student t-test. N, RYGB=4, Sham=4. p>0.05. (B) Average weekly body weight in (g) of pre-operative weight of RYGB-operated DIO *Clock*$^{\Delta 19/\Delta 19}$ mice and wild-type (WT) littermates maintained on a 60% HFD diet and under normal light-dark conditions, compared with their sham counterparts. Mean± SEM. WT Sham n=11, WT RYGB n=10, *Clock*$^{\Delta 19/\Delta 19}$ Sham n=11, Clock$^{\Delta 19/\Delta 19}$ RYGB n=10. One-Way ANOVA followed by Brown-Forsythe test. $^{\&,\$}p < 0.05$. $^{\&}$WT sham vs *Clock*$^{\Delta 19/\Delta 19}$ Sham, $^{\$}$WT RYGB vs *Clock*$^{\Delta 19/\Delta 19}$ RYGB.

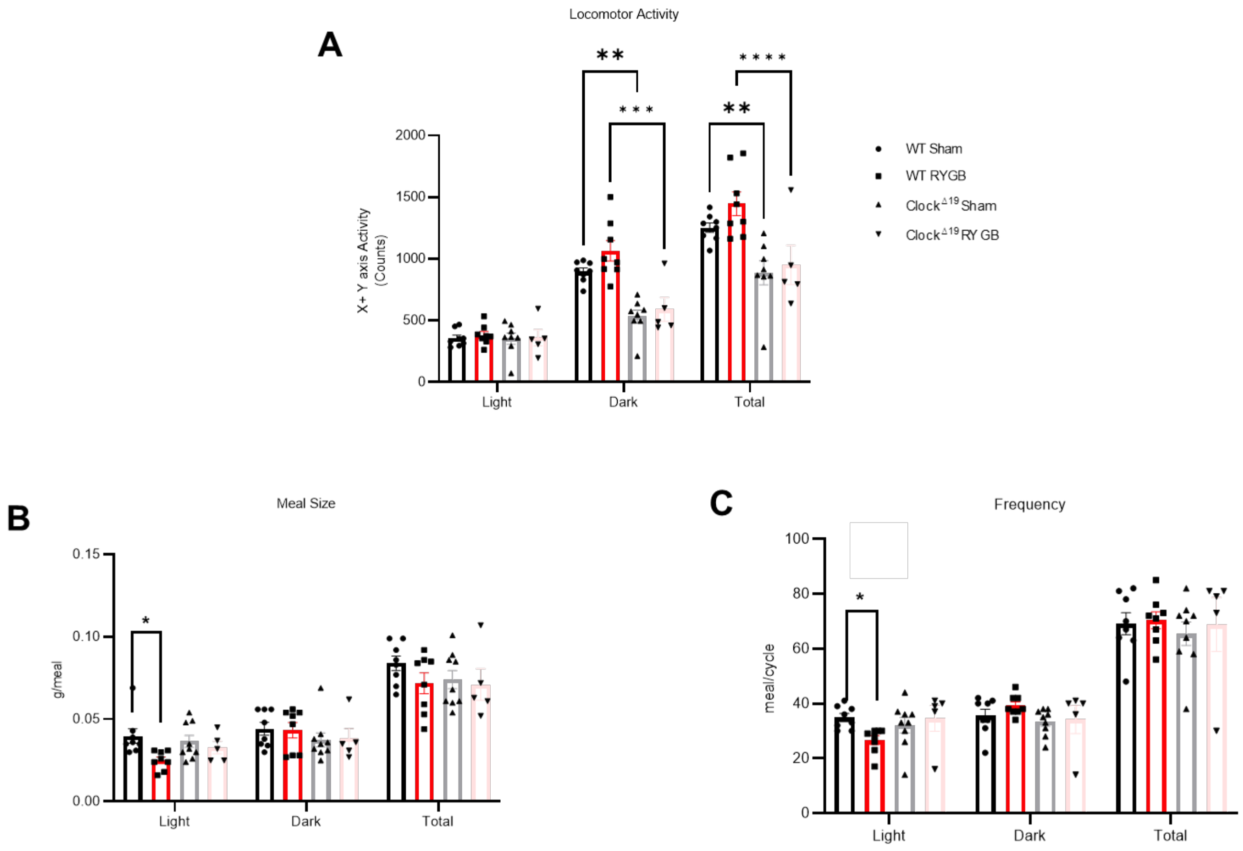

Supplemental Figure 3. (A) Average locomotor activity in (counts or beam breakage), (B) Average meal size in (g/meal), (C) average meal frequency in (meal/cycle) in RYGB-operated DIO *Clock* $^{\Delta 19/\Delta 19}$ mice and wild-type (WT) littermates maintained on a 60% HFD diet and under normal light-dark conditions, compared with their sham counterparts. All data were obtained from measurements taken during the CLAMS system in free moving animals during post-operative week 3. Mean± SEM. WT Sham n=8-11, WT RYGB n=7-10, *Clock* $^{\Delta 19/\Delta 19}$ Sham n=11, Clock $^{\Delta 19/\Delta 19}$ RYGB n=7-10 (clams data n =4). One-Way ANOVA followed by Tukey's test. *, $p < 0.05$, ****, $p < 0.0001$. *WT sham vs WT RYGB.

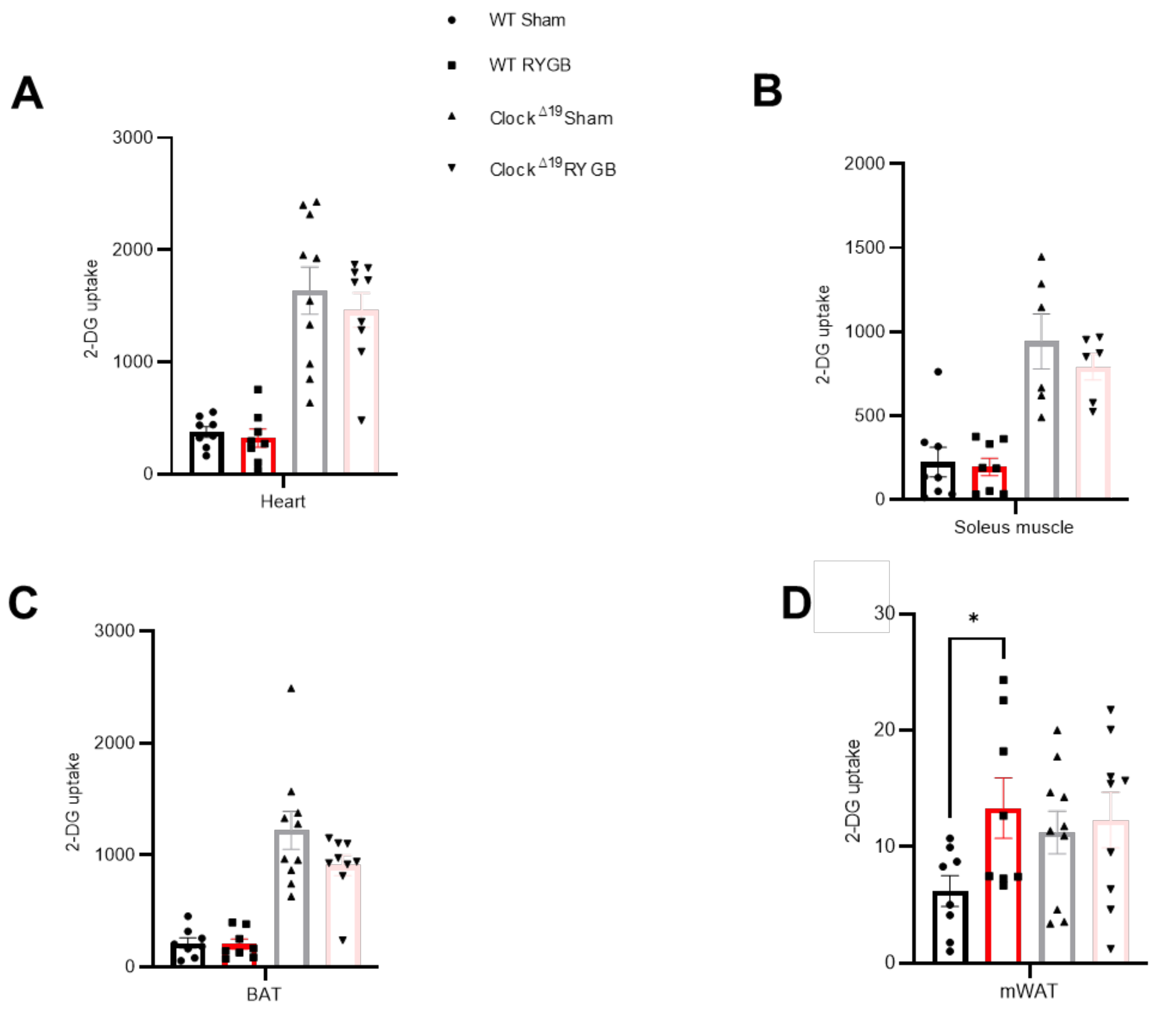

Supplemental Figure 4. Average 2-Deoxyglucose (2-DG) uptake within the (A) Heart, (B) Soleus muscle, (C) Brown adipose Tissue (BAT) , and (D) mesenteric white adipose tissue (mWAT) in RYGB-operated DIO *Clock*$^{\Delta 19/\Delta 19}$ mice and wild-type (WT) littermates maintained on a 60% HFD diet and under normal light-dark conditions, compared with their sham counterparts. All data were obtained from measurements taken during the hyperinsulinemic hyperglycemic clamp six weeks after surgery. Mean± SEM. WT Sham n=11, WT RYGB n=10, *Clock*$^{\Delta 19/\Delta 19}$ Sham n=11, *Clock*$^{\Delta 19/\Delta 19}$ RYGB n=10. One-Way ANOVA followed by Tukey's test. *, $p < 0.05$. WT sham vs WT RYGB.
